# The Introduction of Oral Prophylaxis Treatment into Dental Practice

**Published:** 1911-08-15

**Authors:** Robin Adair

**Affiliations:** Atlanta, Ga.


### THE INTRODUCTION OF ORAL PROPHYLAXIS TREATMENT INTO DENTAL PRACTICE.

BY ROBIN ADAIR, M.D., D.D.S., ATLANTA, GA.

Read before the Tennessee State Dental Association, May 24, 1911.

Several years ago the Dental profession was confronted by the fact of one of its members, a competent dentist, a social favorite, a refined and cultured gentleman, being blacklisted from membership in a swell social club for no other reason than that he was interested in Prophylaxis and that he "cleaned teeth."

Nor was this stigma on Prophylaxis confined to the laity, for again and again at our meetings we heard the statement that it is beneath the dignity of a dentist to clean teeth and it is relegated to the assistant or to the Dental Nurse. Others tell us that "It takes a crank to work Prophylaxis." And when these cranks have appeared before conventions with their sometimes too extreme enthusiasm we have been confronted with humiliating scenes.

The air of mystery has been thrown about these treatments by some of our leaders. Results have been gladly shown, but when it came to the way of doing it, that was another thing, so that the general pracitioner does not get at the root of the matter.

In view of these facts, it is with a degree of hesitancy that I appear before you with a paper on this subject. I too have suffered criticism for my interest in this subject, and I take refuge behind this definition of a crank, that he is one who thinks so deeply upon a certain subject that he becomes a nuisance to all who do not think as deeply as he does.

### SOME INSIGHT AS TO THE HISTORY OF PROPHYLAXIS.

Dr. Levi C. Taylor of Hartford, Conn., wrote me on February 7, 1905, "I find upon investigation that 'prophylactic' means a medicine or medical treatment, the word being very old. In this connection, Dr. M. L. Rhein took exception to this and claimed it to be from a word he gave to a tooth brush in 1882. Prophylaxis came into use in the sixties and was defined by Donaldson in 1874 very much as I defined it at your meeting "surgical or manipulative treatment for the preservation of teeth." That both are a treatment but that each has a proper meaning no one will deny. I believe man and woman both belong to the human family but who would think of interchanging these as meaning one and the same thing."

Dr. M. L. Rhein wrote me a letter on June 10 in which he said, "I don't believe it makes much difference whether you use the word as an adjective or a noun; what I said in Washington was that I was the first person to introduce the word into dental nomenclature when I introduced the prophylactic brush in 1883 and having first made use of the word in that sense, I thought its very use, by virtue of priority, entitled it to be used in that way."

Dr. D. D. Smith writes me on June 10th that Dr. Rhein is entitled to no credit for original work in this matter. He never heard of it or thought of it until I published my first paper in 1898. "Prophylactic" is a remedy and should only be used adjectively. Prophylaxis is never used as an adjective but as a process or description of a process. Prophylaxis is not a preventive remedy but a preventive process. You will find these terms used interchangeably in the Dental Nurse paper and without any discrimination."

On June 18th Dr. Rhein again wrote me "that not one patient out of 500 would understand your purport, although they may declare that they do. I find it is necessary to impress these truths upon them again and again to make them understand them. I don't care a rap about what I call this treatment to my patients. I believe that what they can understand most plainly is the proper term to use, therefor I never say Prophylactic treatment or Prophylaxis to them. Plain English is the best thing to use at all times with a layman. Therefore, I tell them that the cleaning and polishing of their teeth and massaging of their gums done frequently is the best preventive treatment that we have in dentistry. It is all very well to use these words professionally, but plain English is the thing for our patients."

Leaving each individual to take his choice between these opposite opinions and omitting any and all special methods of treatment, I shall at once introduce my subject by the statement which I believe will be generally accepted; that nearly all our dental operations are necessitated by unclean and infected mouths. Then it is not strange that we as dentists have failed to keep those mouths clean? Is it not strange that we have treated this abscess, filled this tooth, operated for Riggs Disease, but think it beneath us to clean the mouth so as to prevent these operations? I know there are many here who will say that they have practiced cleaning teeth all their professional lifes, and that these things will happen any way. But the fact remains that you are mistaken for a thorough search has been made of all available dental literature and no mention of systematic Prophylaxis treatment was made up to the year 1898. About this year two prominent dentists began to investigate these infected mouths and to publish their views and results, but few dentists took up the work. In public exhibitions the actual results were shown by submitting patients who had been under prophylaxis treatment. Drs. Kirk, Darby, Holly Smith, Brockway, Mills and others were enthused and wrote of what they saw but the work went no further until the investigators nearly gave up hope and as one of them expressed it, "went home tired, despondent, and with a feeling that he had done his best and that as the dental profession had repudiated his work, he would make no further effort." But he kept at it and evolved the system of Prophylaxis treatment on the principles of etiology that has accomplished results that have forced us to see the wonderful possibilities there are in store for us, until we now see the dental journals teeming with some new phase in every issue.

Our study and practice up to the present time has been etiology and restoration. It must of necessity be etiology and prevention. Until Oral Prophylaxis treatment was accepted and understood etiology was the subject that Dental Authors wrote volumes and spun theories that now seem ridiculous when we meet them in reading. Detail would make this paper too long and I shall confine myself to facts that have been established beyond peradventure.

1st. That the etiology for the larger percent of dental operations is traceable to local infection.

2nd. That tooth decay is from without and caused from constant immersion in an infected environment.

3rd. That diseased gums whether simple gingivitis, Riggs disease or enlarged glands are rarely traceable to constitutional causes as uremia, or syphilis, but generally to an infected mouth.

If you accept these well established truths I may hope for your interest in the remaining part of this paper. The Medical profession has just emerged from a transformation of its methods from an "all treatment to prevention and sanitation, instead of giving all its time to the treatment of malaria. Physicians now turn to the cause and by prophylactic measures or sanitation seek the death of mosquitoes. The up to date physician now watches the surroundings of his patients to prevent typhoid fever. He takes all precautions to prevent small pox, scarlet fever and diphtheria. "To cure is the voice of the past, to prevent is the Divine whisper of to-day."

Some years ago Dr. J. W. Wassell before the Chicago Odontological Society said "We have been remiss in our duty for half a century in this important branch of prophylactic treatment. The subject before us may seem trite, but we have only to contemplate the situation as it really exists to realize what an important factor the well being of the mouth exercises upon the mortality rate. In order to progress in this or any other direction it is necessary to keep the subject before us. We must realize how much depends upon us as advisors to our patients. It is culpable and dishonest to maintain an attitude of indifference to the principles of oral hygiene, the general observation of which would exert so tremendous an influence in promoting public health."

"The situation demands a quickening of interest on the subject among Dentists. They of course can do the greatest good. Local and National Dental Societies should take the matter in hand officially. Regular reports might be made recommending the best means and measures to be employed in personal care of the teeth. They should disseminate such printed rules as might be approved and secure their incorporation in text-books used in the public schools and colleges, and perhaps make proper use of newspaper and magazine articles, and lectures to the public."

The paper from which this extract came was widely discussed by some of the greatest men of the profession, among them being Dr. C. N. Johnson, J. G. Reid, A. A. Royce, Trumen W. Brophy and A. W. Harlan, all of whom endorsed the movement and wished the public to learn more of this new field of work. One of them said, "I appeal to this and other Societies to publish instruction that can be placed in the hands of patients and the public. Coming from a society it would have more weight than from any individual booklet. Societies discuss these questions, pass some resolutions, but do not act. I would that I could be the means of having this Society publish such a paper. It would be of more importance and value, as a means of reaching not only our own patients, but the public and other peoples patients, than anything we could undertake."

Another one said, "There is a great lack of intelligence on the part of the public, as well as a seeming lack of intelligence on the part of the average practitioner in dentistry, in informing the public as to the best methods of caring for the teeth. In my own practice I come across people who have been patients of dentists from all over the country, men who are supposed to be well up, and yet they do not know the first rudiments of cleaning the teeth. I should be very glad to see this Society put forth some book as has been suggested and place it where it would reach the general public."

Two leaders in this great work of prophylaxis, Dr. M. L. Rhein of New York and Dr. D. D. Smith of Philadelphia both believe alike that this is the most important part of a dentists work but they differ decidedly as to how to put the work into execution.

Dr. Rhein claims that all patients should receive the benefit, and that if he did the work he would have little time for anything else and the charges for the treatments would be a burden to the patient to pay from $5.00 to $15.00 per hour for twelve treatments in the year. He contends that the work is not so difficult but that an assistant can learn to do it in a short time, and he has introduced the "Dental Nurse" into practice, whose duty it is to perform this work for his patients at a nominal charge.

Dr. Smith on the other hand, won't agree with any of Dr. Rhein's ideas and contends that this Prophylaxis treatment is the most difficult thing that the dentist can be called upon to perform, that in so much as it is the best thing that a dentist can do for his patients, and that it takes a great amount of skill, the patient should not go into the hands of an incompetent, but that the dentist must charge accordingly and do the work himself.

Having determined to introduce this work into our practice the next thing was how best to accomplish it. It took Dr. Smith one year to induce 25 patients to submit to the treatments. Others had an equally hard time in the beginning. Dr. Crouse said he could not get his patients to come at regular intervals.

Believing that we had many patients who would appreciate an effort in this direction if they only knew of its advantages I set out to let them know. Some of these patients I would not see for six months. To take the time and call up each one and take from 15 to 30 minutes explaining the system was out of the question. If I started the work I must have a small number at least to make the work interesting. The only practical way seemed to send these patients some literature and I attempted to get reprints from some reputable dentist but failed to get that which would answer the purpose, so I prepared a simple pamphlet describing what the treatment was and what I had seen it accomplish. The pamphlet was only a temporary expedient. It contained truths that I wanted my patients to learn, there was no advertising in it or on it and if it fell into the hands of the patients of other dentists it did not come from me and would do them no harm. I was criticised by some of my special friends for sending out this pamphlet. Dental ethics should be upheld but if our code prevents us from spreading knowledge among ones patients it should be amended. An extract from the April "Dental Digest" on "Ethics" reads "Our duty to our patients is considered first," which is right. However great our love for, and devotion to our profession and its members may be, the greatest good of the patient should be our first consideration. Anything short of seeking the best methods, and rendering our best services is unethical. Anything that tends to educate the dental patients along dental lines should be encouraged especially if it be on the subject of oral hygiene.

So after experiencing all kinds of difficulties we finally persuaded or almost forced 30 patients to submit to the "terrible ordeal" (?) of having their mouths attended to each month. The plan that I adopted was to set aside a certain day in each month. Mrs. Smith's appointment was on the 10th of each month and she was reminded the day previous to be on time; and so on with the list. The usual ridicule by a few of my dental friends of my "advertising scheme" as they called it soon had the effect of swelling my list to such an extent that I had either to turn the work over to an assistant, thin the ranks by charging more, or to get up some scheme by which I could continue the work at a nominal price to the patient, or lose nothing myself.

I decided to devote certain days in each month to this work, and decided on the second Tuesday, second Thursday, second Saturday, and the second Wednesday in each month. Mrs. Smith's engagement was changed from the 10th to 8 A. M. on the second Thursday. At 8:30 o'clock I expected Mrs. Jones and so throughout the day, treating from 12 to 20 patients on each one of these days. The office maid attended to the reminding and as the charge is entered for a year, failure to meet these engagements results in loss to the patient. It in no way interferes with my regular work of restoration for those patients who are irresponsive to my efforts to save them much of this expense pain and trouble.

The scheme enabled me to give to my patients this sweet charity, for I doubt whether the days work of prophylâxis would more than pay expenses and I am sure that it reduced my income one-fifth anyway, as these patients were the cream of my practice and few of them have had new caries and their mouths are in such healthy condition that other dental operations are unnecessary. The reason for this pecuniary deficit that I experienced is well explained by giving an extract from a letter written to me by a prominent surgeon, a man who would get from $50.00 to $200.00 for an hours surgery. He wrote me, "Before patronizing you I had been getting my teeth cleaned by reputable men from $1.00 to $1.50 and the only reason for transferring my patronage was my friendship for you and my inclination to help young men. But I do not think my pocket book is commensurate with your new prices and I am glad and think you are to be congratulated if you can get prices above the older men in your profession."

If I am reliably informed many prominent dentists do this work for nothing and sometimes they get as much as

50 cents, though they won't admit the charge to a professional brother. But seriously the scheme of devoting certain days in each month will enable the dentist to introduce this important work at such a cost to the patient that hé won't feel it. Do it for nothing if you can't get them any other way. You will soon get them interested and good results will follow. You will get more than you can attend to and then dismiss those who will not pay a reasonable fee.

### DENTISTS AND THEIR MOUTHS.

We cannot teach others what we do not know or practice ourselves and Dentists are the first who need this instruction. What a record it would make if the mouths of every dentist here were inspected at this moment! I can tell you without examination, for I recently visited the office of 20 prominent Dentists, interviewing them on their personal care of their teeth and to see if they were making any attempt at introducing any systematic prevention treatment. The following table gives the result:

Dentist No. 1. Didn't care anything about the matter, refused to talk or let me examine his teeth. To my certain knowledge this man has an unclean mouth and is loosing his teeth by Pyorrhea.

No. 2. Ex-Dean of a dental college. Did not think it a good idea to write on the subject. Refused examination but admitted that his mouth was in bad shape and that he did not have time to get his teeth cleaned.

No. 3. Uncleanly mouth, bridges incrusted with scum, tartar on lower teeth.

No. 4. Many fillings, also bridges, whole mouth in fair condition. Gums firm, was chewing tobacco while operating. Some attention paid to cleaning teeth.

No. 5. Mouth was extra clean, teeth polished and gums firm. Believes in prophylaxis but has no regular treatment. This dentist was a lady.

No. 6. No attention paid to Prophylaxis. Pyorrhoae.

No. 7. Many fillings, wears plate. Unclean mouth. Riggs disease. Stranger to brush.

No. 8. Few fillings. Clean mouth made so by constant attention. Some attention paid to prophylaxis.

No. 9. Clean mouth. Believes in clean teeth but does not practice prophylaxis.

No. 10. Uses tobacco, teeth in fair condition.

No. 11. Tartar present but brushes his teeth twice a day.

No. 12. Unclean. Riggs Disease.

No. 13. Too busy to give me an interview of 2 min.

No. 14. Mouth in good condition. Says that he hasn't time to practice prophylaxis.

No. 15. Mouth in good condition.

No. 16. Always cleans teeth before operating. Has some twenty patients who report to him for this work every three months. Gets good fee for it too.

No. 17. Mouth in good shape. Assistant does the cleaning. In accord with the movement and will do more from now on.

No. 18. Good condition.

No. 19. Unclean mouth.

No. 20. Fair condition.

To summarize, I find that most of them chew and smoke, some of them while operating for ladies. There are 7 dentists out of twenty with a fairly clean mouth. Six were afflicted with pyorrhoea. Only one was making any effort toward systematic measures for prevention with his patients.

### TECHNIQUE.

If you should ask me my method of treatment I will tell you that I have none except to scrub and polish the teeth and massage the gums. I use the word "scrub" advisedly although I know that Dr. Smith will hold up his hands in horror when he hears the word. As long as you handle so gently that tender gums that bleeds at the slightest touch, as long as you look upon teeth as a fragile structure that will be harmed if polished, you will fail at prophylaxis treatment. No man ever possessed a clean mouth by gentle handling of the brush and water alone. On the other hand no person ever had his teeth injured by any good dentifrice or mouth wash. All I have to say is that the person who thinks he keeps a clean mouth by simply brushing the teeth is greatly deceived and the dentist who thinks the same thing has never seen a perfectly clean set of teeth that have been under this prophylaxis treatment. Use any method or all of them. Use only the Smith method of hand polishing with orange wood and pumice, if you will do it well. Use any agent that you like that will not chemically effect the teeth. Use the dental engine or not as you like. Only be sure that you polish not clean, but polish those teeth at least once per month. You can get the rust off a piece of iron by cleaning, but it is another thing to polish that iron. When you clean teeth you leave a ground glass surface to which tartar and secretions will adhere, but not so when you polish them on every surface.

### EDUCATE YOUR PATIENT.

Another very important item to be considered in this scheme of mine is for the dentist to TALK, TALK, TALK all the time while doing his work. Not of theatres or receptions but of the importance of the patients attending to his mouth, what you have accomplished, what you hope to accomplish and what the patient must do to assist you. Report the same thing at the next sitting for in a months time he has forgot it all and it will be new information to him. I often turn down the pages in dental journals at papers and discussions on prophylaxis and should I have to keep a patient waiting, they are requested to read these articles and to discuss them with me. In about three months even the uncouth, gawky, rough, don't care boy of 15 years will get interested in his teeth and you will have smooth sailing from then on.

Before a new patient is permitted to enter for treatment they should have their mouths cleaned up in the usual way by removing all deposits and all cavities filled. For prophylaxis pre-supposes a clean and patched mouth to work upon. This work should be done on regular operating days and the usual charge made for same.

### RESULT, WORK.

Patients now present themselves each month and we feel greatly encouraged to have these mouths free from sensitive teeth and gums, devoid of all diseased conditions, and almost immune from decay. This changed condition has been maintained until the general health has been greatly benefited as it has prevented the introduction into the stomach and lungs of a lesser quantity of toxic products. These teeth present a polished appearance that add to the looks of their possessors, and will prolong the life and usefulness of the teeth.

Other Dentists are accomplishing these results. Why not all of us?

I plead for you as dentists to have your teeth polished by your fellow practitioner so that you may know personally the joys of a clean mouth, then you are in a position to impart this knowledge to your patients.

I plead for action as well as enthusiasm. I would that we could get out such a pamphlet as would explain to the laity what this treatment means and that every dentist would do something toward educating his clientele.

I plead for you to devote one, two or three days in each month to this work. You will not miss the time, and it will repay you in knowledge gained and satisfaction of duty done, if not in dollars.

Let us arouse some genuine interest and act in such a way that we can go forward in this work that will prolong the life of the dental organs, and add to the looks and health of mankind.

DISCUSSION.

Dr. H. W. Morgan: I can but re-echo the words of the president in congratulating Dr. Adair and the Association on having him with us, and for his having gone into the history of the subject, particularly that which led him to take up the work. And one thing that appeals to all of us first in listening to Dr. Adair—those of us who are acquainted with what he has been doing and of the system and methods that he has been preaching in his practice knew it before he came here—to what extent he had reorganized and re-arranged his practice that he might carry out what he has outlined to us to-night. Without that system and without his trained analysis as a matter of course he could give but little of his time and expect to receive but little result from his efforts.

Personally I have much preferred, and use the word oral hygiene. I think it preferable, as Dr. Rhein suggested, that it was his opinion that as it applied more directly to people than the other it was a better term to use. There isn't anything that we do in the dental chair for our patients that is not prophylactic; consequently if you want to specialize and get away from the treatment of diseases and do something that is preventive, then it becomes hygienic, and I think it will appeal to the medical man with greater force than the term "prophylaxis."

The essayist intimates that the time is coming when the practice of dentistry along the old lines of etiology and *restoration*, will be along the line of etiology and *prevention*. We are all of us willing to accept that. There never was in the history of the world a time when so much attention in current literature, not professional, could be found on almost every subject dealing with the welfare of the human body, or human race, we might say. The world seems to be interested as never before in this subject. Because since the period of 1880 to 1890, such wonderful progress has been made in medical knowledge and of the causation of disease the world has awakened to the fact that some diseases which seemed to be incombatible, can not only now be cured, but can be prevented, and it is this phase of it that is appealing to humanity. Consequently when we come along with our claim that we can prevent caries and pyorrhea the world is willing to listen to us. Not only the dental and the medical profession, but the laity themselves have been aroused to the importance of it. I do not believe thirty years ago Dr. Adair could have ordered his patients around for twelve treatments a year, but it is because we have reached this condition in medical knowledge that enables him to accomplish so much. That much has been done and that much can be done, I am ready to admit. I am glad to find that while confessing himself to be a crank, he is much less of a crank than he seems to be, for he takes a rational view of it. For instance, he uses such words as "almost," and at times he says he believes that we are going to accomplish, thus and so, in the future, not that it all can be done. Many of our oral prophylaxis cranks claim that it can always be done; but there are some things that I am not willing to admit. There are structural imperfections in the teeth, that, until we can advance in the physilogical development of their structure, we can not expect to shut out caries. Caries is one thing that we cannot measure and control, yet until we can meet this condition all our prophylaxis must fail at that point. I want to close my remarks with an endorsement of the dental nurse. I believe it is the solution not only of this question in dental offices, but I believe that the dental nurse in our schools is going to be the solution of the question of what we shall do to educate the people. When we can persuade our boards of education in every school in the land to place in our schools a trained nurse, and that trained nurse is going to be trained not only in medicine but in dentistry, to point out defects, not only the defects of the teeth, but defects in the eyes and the ears. The pale-faced women suffering from tuberculosis, and the pale-faced children with something radically wrong with them, physically and the women who are teaching in our schools when they are lagging in their work, and the principal has not the courage to say you must step aside and let a normal teacher do your work, all this must the trained nurse of the future do. The trained nurse must know all of the propaganda for oral hygiene as well as the examination of the pupils.

Dr. R. Boyd Bogle: I want to add my commendation to what Dr. Adair said. He admits that he has gone through the mill, has been through the mill for about eight years, admits that he is still in the mill, and I expect if you saw him about a year from now he could tell us a whole lot of things he has found out from his experience, and I am going to take the liberty of inviting him to come back next year. His talk shows conclusively that he has put a great deal of thought and study on this subject. While oral prophylaxis is in its infancy, still I believe there is a great deal that can be accomplished even now. One thing in particular I want to refer to is the system with which he goes about his work. I believe if we do not have something definite to accomplish and some definite way of accomplishing it, then we do not get much out of it, we do not get results.

Dr. Stanley Rich: I heartily agree with Dr. Adair in all of his statements, and I want to say that while attending the meeting of the Southern Branch at Atlanta recently I had the pleasure of seeing one of Dr. Adair's patients who has been taking his treatment for some time, and I can truthfully say I never saw a mouth in a more beautiful condition. That one case was sufficient to convince me that great things can be accomplished by this treatment.

Dr. W. C. Gillespie: Everything I heard the essayist say was good, except the statement that he was afraid to give out anything with his own name signed to it. Now any man that has got sense enough to give us what he has given us to-night, and do what he has done in this work, has got sufficient intelligence to warrant him in putting his name to anything he wants to write, regardless of what any number of bone heads might feel called upon to say under the cloak of ethics, when it was merely jealousy and envy. Anything with Dr. Adair's name signed to it would get my attention any time after what I have heard of him. He can very greatly aid the work by putting his name to it, a few of them can grouch around all they want to, but he has delivered the goods, and if the other fellow does not come up to the standard, do not pay any attention to him. In his use of the vibrator I think he has got one of the best instruments that is in the hands of the dentists to-day, in the treatment of such troubles as he has outlined. The vibrator goes a great deal further in producing results than would be imagined by a man who had never used one. We hear a great deal of the massage of the tissues with the fingers and various instruments, whereas the vibrator goes through all the tissues involved in these surfaces and penetrates all the way through, just like a vibration would do if you threw a rock into a pond. By placing it against the crown of the tooth you massage the peri-dental membrane, and its results in my hands along that line have been marvelous. I want to emphasize the use of that vibrator if you want to get results. Another thing that commends the Doctor's paper to me is that he is not tied down to any special method of using his instruments, or any particular set. He gave us to understand that he used whatever will produce the results desired; and a man who can't use but one set of instruments must labor under a great handicap. If he has got to have a certain instrument deflected this way or that before he can do it, that man is a very poor manipulator of instruments. The use of hand instruments in polishing teeth is good, but the use of the dental engine with its various attachments has got to be governed by the necessities of the case, and I certainly think more of a man's theories who can show that the same thing can be done in a number of different ways. The important thing is to get the result instead of using a certain instrument. I certainly think a great deal more of that man and his theories than of the man who can do it just by one set of rules.

The paper is instructive to us all and should be encouraging to us all. Many say, you can do this and that, you can't do that, or you can't control your patients, and it is all a mistake. You can do what you are determined to do. If you let your patients boss the job, you will do what the patient is inclined to do, but if you boss the job, the patient will do what you suggest. If you talk to them in a domineering or dictatorial way you rouse their antagonism, and you can do very little with them. But if you talk to them in the way that convinces them they will come very near doing what you want them to do.

Dr. Meadows: The essayist has suggested in the first place that we begin to clean up with ourselves. I remember at a meeting in Birmingham, I don't know whether it was the Doctor, his father, or some other member of his family, was making a wager that there was not a clean set of teeth in the audience. I began to duck, because I had left my tooth brush at home, I knew my mouth wasn't clean, and I took my dental engine and polished my own teeth before this meeting convened, besides my daily use of the brush. Now I believe in the first place, before we begin to preach prophylaxis, we ought to begin at home and do a little housecleaning. It would be well for us to go home and clean up ourselves and then preach dental prophylaxis. He has suggested to us that there is a system in cleansing teeth and maintaining oral prophylaxis as well as in doing any other work in our line, and there is, and we should adopt a system.

Dr. J. W. Winn: I saw Dr. Adair's clinic in Atlanta, and I saw his patient, one that he had treated for several months I believe; her teeth were absolutely clean so far as I could determine. There was a perfect luster on the enamel of her teeth just as though they had been polished like your finger nail. I think the doctor had used some sort of polish such as the oxide of tin. Since coming home from that meeting I have been using that same flour of pomace and oxide of tin.

Dr. Meadows: I would like to ask Dr. Adair before he closes this subject to give us some idea regarding the discussion which has been published in regard to the use of the rubber cup and bristle brush. As a matter of fact I do not make use of the rubber cup in polishing the teeth, but I do use the bristle brush, what is known as a Robertson brush. I get better results from the use of the Robertson brush than I do from the rubber cup. I abandoned the rubber cup several years ago, and if I am wrong I want some one to set me right. I want to know why it is that some advocate the use of the rubber cup, and some use only wood points, and never use the bristle brush.

Dr. Noel: I want to ask the doctor what he thinks about this; in examining my patients' mouths I find almost invariably that one side is in a better condition from a hygienic standpoint than the other, it may be the right, it may be the left, the general rule is that the cleanest side is the side upon which the food is masticated. I try to impress upon my patients the necessity of using both sides of the mouth. You know the parts of our body to grow must receive exercise, and the same is true, though not to such an extent, of the teeth.

Dr. Jones: I would like the Doctor to tell us how he instructs his patients to use the brush. I have my own idea about the way a patient should use a brush, but I

would like to know how he uses the brush, and see how we co-incide.

Dr. J. R. Beach: Mr. President, the cleaning and polishing of the teeth is an operation with which we all thought we were familiar. No doubt there are many here, or a few at least, who are not familiar with the technique of this work. Why do you clean and polish teeth? It is for preventive measures. In medicine it is believed the time is coming when there will be no occasion for so much attention to the curing of diseases, and it is a fact that important advances have been made along that line, where preventative measures have been used, thereby doing away with the necessity of curing the disease when it does not necessarily have to exist. Now Dr. Adair I think has opened the eyes of a number of us here who thought we knew about as much about cleaning and polishing teeth as any one could. It is an important matter, and you may skip over it if you please; turn it over to your assistant or a nurse, or whoever you may have in your office to do that work. If it is delegated, you should be assured that it is done properly. I think it is an absolute necessity for every dentist to give this matter his best attention. When Dr. Adair started out he made some remark about a crank and gave a definition of a crank, as that which turns something; and he makes good on his definition as he turns something. He ought to turn the minds of those present here to-night into the channels of better cleanliness of the oral cavity.

Dr. Leonard: I want to express my appreciation of the paper and my pleasure, and to say that I had no idea that the subject had so much in it. I do not remember whether the essayist mentioned the use of nitrate of silver as a prophylaxis treatment. I have heard some others speak of using nitrate of silver in dilute solution for this method of so-called prophylaxis, the solution not being strong enough to cause discoloration, and I would like to ask if it is best to use this solution, and just how strong he applies it. I would like also to have his mode of treatment of the erosions that we so often find at the cervical margins of teeth, conditions that are very difficult to treat by the methods that I have been able to employ.

Dr. Adair: Of course I can't possibly answer all these questions, it will take too much of your time. I appreciate more than I can tell you the complimentary things that have been said. What I am driving at is not my personal doings, but I want you to go home and do this work, then I will go back with a much better feeling than you have created by the nice things you have said about the matter.

The first thing is about silver nitrate. We do not find its use necessary because the mouth has been put in perfect shape.

As to the cases of erosion, your own Dr. Noel here in the city has written that up several times, we simply polish it, take off the rough places, and in severe fissures we cut them out. You will notice that they do not run through the enamel. Cut them out so your brush will go into them. In children, you can fill these fissures up with cement until the case gets old enough to handle by the other method.

As to different sides of the mouth, years ago prophylaxis was not so much needed because we chewed our food longer, and had rough things to eat. To-day we do not chew our food, we bolt it.

As to brush and cups. Don't ask my particular method. You can take either or both. I too, Dr. Morgan, agree with you as to the use of the term "Oral Hygiene," but you have got to use some dignified kind of name, other than cleaning teeth in order that it may be respected.

Now as to your schools, you have got an opportunity here in your state and in the city of Nashville to do the greatest work imaginable. Tennessee is an educational center you might say, and if you men will go to your school authorities and approach them in the proper manner, you will get oral hygiene in your schools all right. The dental nurse is the solution of this problem in the schools. And your nurse is expected to prepare your patients for operation. If I go to the hospital I expect my patient to come into the field of operation cleaned up, and the dental nurse is the one to do it.

As to the technique, I am going to leave that board up there, it will take too much of your time to explain all the different methods of work and the instruments used in them. I had a patient whom I had been treating, and had given her a brush and instructed her how to use it and she came back to me with her mouth in a pretty bad condition, and I told her that she hadn't brushed her teeth as I had told her, and I gave her a brush and asked her to brush her teeth just as she did at home. Gentlemen, she brushed her teeth as pretty as you ever saw anybody, but she had not touched the gum margin at all. In the first place I give them a brush and ask them to brush their teeth and ask them if that is the way they brush their teeth at home, and then I show them how to use the brush, and show them how to go around the gum margins and up in the roof of the mouth, tell them to get away up under it.

